# An Integrative Analysis of Meningioma Tumors Reveals the Determinant Genes and Pathways of Malignant Transformation

**DOI:** 10.3389/fonc.2014.00147

**Published:** 2014-06-23

**Authors:** José Carlos Iglesias Gómez, Adrián Mosquera Orgueira

**Affiliations:** ^1^Independent Video Editor, Santiago de Compostela, Spain; ^2^Independent Researcher, A Coruña, Spain

**Keywords:** meningioma, cancer, whole-genome, integrative analysis, transcriptomics, malignant conversion, video presentation, no funding research

## Abstract

Meningiomas are frequent central nervous system neoplasms, which despite their predominant benignity, show sporadically malignant behavior. Type 2 neurofibromatosis and polymorphisms in several genes have been associated with meningioma risk and are probably involved in its pathogenesis. Although GWAS studies have found loci related to meningioma risk, little is known about the factors determining malignant transformation. Thus, this study is aimed to identify the genomic and transcriptomic factors influencing evolution from benignity toward aggressive phenotypes. By applying an integrative bioinformatics pipeline combining public information on a wealth of biological layers of complexity (from genetic polymorphisms to protein interactions), this study identified a module of co-expressed genes highly correlated with tumor stage and statistically linked to several genomic regions (*module Quantitative Trait Loci, mQTLs*). Ontology analysis of the transcription hub genes identified *microtubule-associated cell-cycle processes* as key drivers of such network. mQTLs and single nucleotide polymorphisms associated with meningioma stage were replicated in an alternative meningioma cohort, and integration of these results with up-to-date scientific literature and several databases retrieved a list of genes and pathways with a potentially important role in meningioma malignancy. As a result, cytoskeleton and cell–cell adhesion pathways, calcium-channels and glutamate receptors, as well as oxidoreductase and endoplasmic reticulum-associated degradation pathways were found to be the most important and redundant findings associated to meningioma progression. This study presents an integrated view of the pathways involved in meningioma malignant conversion and paves the way for the development of new research lines that will improve our understanding of meningioma biology.

## Introduction

Meningiomas represent approximately a quarter of the total central nervous system (CNS) neoplasms. These tumors are derived from normal arachnoidal cells of the leptomeninges, appear tightly joined to the *Dura Mater*, and tend to be located along the parasagital sinus, over the cerebral convexity, in the sphenoid wing, around the pontocerebellar angle or along the dorsal region of the spinal cord. Although as a group they are considered to be benign, variability in recurrence frequency, life expectancy prognosis, symptoms, and histological appearance exists. In this regard, histological analysis reveals that 80–90% of the meningiomas are benign [World Health Organization (WHO) Grade I], which are not associated with an excess of mortality when totally resected. However, about 5–15% of them are atypical (WHO Grade II) and associated with a marked increase in recurrence frequency and a small risk of death. Only 1–3% of the cases become anaplasic or malignant (WHO Grade III), developing a high tendency to invade brain structures, metastasize, and to recur (1–3). Overall, Grade II tumors have an average life expectancy of 11.9 years and an average recurrence-free survival of 142.5 months vs. 3.3 years of average life expectancy and 39.8 months of recurrence-free survival for Grade III meningiomas (4).

Ionizing radiation exposure is the principal modifiable risk factor. Age and female gender (especially during the reproductive period) are known non-modifiable risk factors. Nevertheless, genetic factors have also been found to play a role in meningioma development and predisposition. Type 2 neurofibromatosis (*NF2*) is an autosomal dominant condition related to a mutation on chromosome 22q12 and is a common condition related to elevated risk for developing meningiomas, among other neoplasms (4). In fact, loss of heterozygosity at several points in the 22q locus has been shown to be an early event in the development of benign meningiomas. Curiously, a study by Black et al. (5) revealed that meningioma gene expression is correlated with progesterone receptor levels, and this is particularly true for those genes encoded near 22q12. Single nucleotide polymorphisms (SNPs) in the *Ki-RAS* and *ERCC2* genes were associated with increased risk for developing meningiomas. Genes involved in detoxification, reactive oxygen species mitigation, metabolism, and DNA-repair also seem to be involved. In this regard, SNPs in the C variant of *SOD3* (superoxide dismutase 3), *GSTT1* (glutathione *S*-transferase theta 1), and *MUTYH* (Muty homolog) have been found to be associated with meningioma risk. Moreover, association of SNPs located in genes related to apoptosis (*CASP8*) and cell-cycle pathways (*GLTSCR1, ERCC4*, and *PCNA*) are also known (1).

Since the discovery of the scale-free property back on 1999, living phenomena have been shown to be organized in networks regulated by this property (6–14). Hovarth et al. (15) have shown that gene expression can be analyzed as partially discrete co-expression networks where connectivity among genes follows the scale-free property. The main aim of this study was to assess the relationship between genomic markers and gene-expression data of meningioma tumors at the network level. The results of this analysis were integrated with protein–protein interaction (PPI) networks, transcription factor binding sites (TFBS), miRNA target sites, and pathways data. Two previous gene-expression analyses and a genotypic study were used for replication purposes. Overall, redundant findings for the implication of several pathways in meningioma progression are described, and a list of potential markers is provided for future experimental validation.

## Materials and Methods

**Figure 1:**
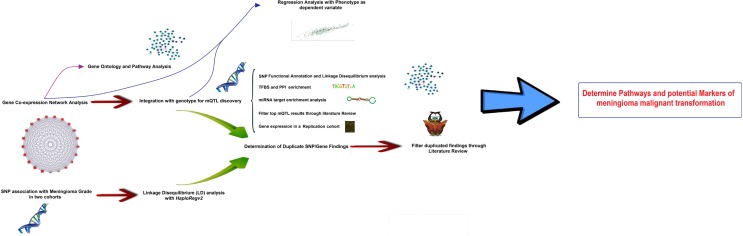
**Pipeline scheme representing all the steps followed in this research**.

The core of the study is based on the integration of genomic marker information (i.e., SNPs) with gene-expression data on a group of meningioma tumors, published by Nelson et al. (16), in the search for genomic loci influencing malignant-like gene expression. In the first part, *Weighted Gene Co-expression Network Analysis, WGCNA* (15, 17) was applied to explore gene expression as an undirected co-expression network and reduce its dimensionality. A co-expression module deeply correlated with meningioma biological parameters was discovered, and the top hub genes inside the module were identified based on network analysis parameters. In a second step, genetic loci associated to the meningioma-related co-expression network were identified, in an approach known as *module Quantitative Trait Loci* [mQTLs (18)]. These loci were found to partially overlap with SNP association with disease stage. In the third step, the most likely causative genes in the proximity of the mQTLs were delimited, which formed the input list for their integration with PPI networks, TFBS data, miRNA signatures, and pathways databases. Multivariate regression models were created in order to determine what extent of the variability in WHO meningioma Grade could be explained by mQTL SNPs and co-expression module data. With the help of literature filtering, a list of genes with a high potential role in meningioma malignant conversion is provided for future experimental testing. A study pipeline scheme can be consulted in Figure 1.

### Initial data source

The initial data input for this study was of a group of 85 meningioma samples [GEO accession *GSE16584* (16)]. Meningioma samples (22 males and 37 females) with both genotypic and gene-expression data were selected. This resulted in a total number of 59 samples, of which 39 were WHO Grade I meningioma cases, 15 were WHO Grade II, and 5 were WHO Grade III (Table S1 in Supplementary Material). Tumor genotyping was performed with the *Human Mapping 100K Set* platform (*Affymetrix*) and gene-expression data were obtained using *GeneChip Human Genome U133 Plus 2.0 Arrays* (*Affymetrix*).

Genotyping *Hind* and *Xba CEL* files were downloaded from GEO [*Gene-Expression Omnibus* (19, 20)]; accession code *GSE16583* (16) and analyzed the *Affymetrix Genotyping Console* software (*Affymetrix*). Only samples with a quality control ≥90% were included. After pre-processing all the data results, we selected 111,829 SNPs, which passed three filters: minimum allele frequency of 5%, minimum call rate of 95%, and evidence of Hardy–Weinberg equilibrium (*P*-value ≥0.01).

### Gene-expression analysis

Expression *CEL* files were also downloaded from *GEO* (GEO accession code *GSE16581*) and analyzed in *R* (21). Samples were read, background corrected, normalized, probe-specific background corrected, and summarized into an *R eset* class object using functions of the *affy* package (22). Array quality was determined with the *arrayQualityMetrics* package (23). In order to simplify the analysis, we applied a filter to select only those roughly 12,000 probes, which showed at least a 1.8-fold expression change according to the median in at least 10% of the samples (24). In order to find *expression quantitative trait loci* (eQTLs), and since no Y chromosome SNPs were measured in the chips, we also excluded gene-expression probes pertaining to such location.

Weighted gene co-expression network analysis functions were applied to expression data according to several online tutorials (18). The adjacency matrix was calculated using a soft-thresholding power of 6, which showed an approximate scale-free topology (*R*^2^ = 0.75). Briefly, the connectivity value of each selected transcript (calculated similarly to kIN in Section “Regression Models”) was used to create a group of 10 bins with equal size, and each connectivity value was assigned to each bin. The connectivity distribution *k* was defined as the average connectivity value for each bin, whilst the probability distribution of *k p*(*k*) was defined as the ratio of the number of connectivity values in each bin by the number of connectivity values studied. Under the approximate scale-free topology assumption, the logarithm of *p*(*k*) (*log(p(k))*) and the logarithm of *k* (*log(k)*) are strongly negatively correlated. In this case, by using a soft-thresholding power of 6 we obtained a *R*^2^ = 0.75 (Pearson’s correlation of 0.86, regression slope of −1.46), whilst using no thresholding at all we obtained *R*^2^ = 0.03 (Pearson’s correlation of 0.17, regression slope of 1.05). Thus, the soft-thresholding value selected ensures approximated scale-free topology whilst retaining a higher number of informative connections in the network. Several co-expression modules were determined, and correlation between phenotypic data and their respective first principal components (a.k.a. *module eigengenes*, ME) was calculated. Mean gene significance with WHO Grade was calculated as the absolute average correlation of all module genes with this trait. Due to its marked positive correlation with several parameters (refer to Figure 5), the pink-module was chosen for further analysis. *Module membership* (MM, *a.k.a kME*) was defined as the Spearman’s correlation between the ME and the genes corresponding to the pink-module, which is considered a measure of centrality of each gene in the network. Gene-expression standard deviation was determined with the function *rowSds*, part of the package *matrixStats* (25).

### Gene co-expression network visualization and pathway analysis

*Cytoscape* (26) was used to create a graphic representation of the pink-module. Using the *CentiScape* plug-in (27), we analyzed several network parameters, such as *degree* and *betweenness centrality*. Hub genes were chosen as those with a degree higher or equal to 250 and a betweenness centrality value higher or equal to 2,500. The *ClueGO* plug-in (28) was utilized to create pathway and Gene Ontology (29) enrichment networks. Gene ontology databases for *Biological Processes, Cellular Components, Immune System Processes*, and *Molecular Functions*, as well as *KEGG* (30, 31) and *Reactome* (32), were included in the analysis. Enrichment analysis was performed with the hypergeometric test, and a significant FDR-adjusted *P*-value threshold of 0.001 was selected. *ClueGO* gene ontology and pathway terms were filtered, so that only those matching to at least 10% of the genes in the pink-module were considered. Gene ontology analysis for hub genes was performed with *DAVID* web tool (33, 34).

### mQTL finding, annotation, and downstream functional analysis

The degree of association between the the pink’s module ME and the genotypes was performed by fitting a logistic regression with SNPs as dependent phenotypes with the function *spn.lhs.tests* implemented in the package snpStats (35). Since the ME does not reflect all the variability of the whole module, the sum of the first and second principal components (*PC1 + PC2*) was also studied in a similar way. Finally, we also tested for significant improvements over the base model of the ME by separately adding PC2 and PC1 + PC2. SNPs with a *P*-value in the order of 10^−5^ or less were selected as mQTLs, and in case that various SNPs mapped to the same gene only the most significant was chosen.

mQTL putative genes were obtained from the HaploReg v2 web browser (36). mQTL enrichment in *TFBS* was studied using *oPPOSUM Single Site Analysis* tool (37). Briefly, the analysis was run with the following parameters: (1) Vertebrate JASPAR CORE profiles were selected, (2) only the top 10% conserved regions (with a minimum conservation of 70%) between the mouse and the human genome were included with (3) a position weight matrix match threshold of 80% and (4) within a region of 5,000 bp up and downstream of the transcription start site for each gene.

Gene Ontology and Pathways analysis for mQTL putative genes was performed with Cytoscape and ClueGo similarly to Section “Gene Co-Expression Network Visualization and Pathway Analysis.” In this case, a threshold of 3% was set to the proportion matching genes in the ontology group. Gene set enrichment analysis (GSEA) was used for miRNA target site enrichment (38). The PPI database STRING (39) was used to test for known interactions among the proteins encoded by the mQTL genes.

To address the possible influence of genes in the pink co-expression module over mQTLs, a search for mQTL putative genes matching to in-module genes was conducted. Finally, the PubMed database was interrogated for known associations between mQTL putative genes and cancer or meningioma (search terms: “*Gene Name AND Cancer*” and “*Gene Name AND Meningioma*”, date: 28/4/2014).

### Gene expression and genotypic replication cohorts

An expression replication cohort was constructed with data from GEO [GEO accession codes *GSE43290* (40) and *GSE4780* (41)]. Matching samples in both datasets were selected, and batches were removed with the *ComBat* function implemented in the package *sva* (42), getting an overall cohort of 22,283 probes and 103 individuals. Linear models of the *limma* package (43) were applied to determine differential gene expression between tumor stages and to detect matches with pink-module genes. Wilcoxon Rank Sum Test was applied to determine possible connectivity differences among those probes found to be overexpressed in the replication cohort compared to its background. Background connectivity was determined as 10,000 times random permutation of kME values for pink-module probes present in the replication cohort.

At the same time, a genotypic cohort of 50 meningioma samples was downloaded from GEO [GEO accession code *GSE42624* (44)], which was used to find overlapping disease progression-associated SNPs/genes in both datasets, as well as to detect matches with mQTLs. Gene overlaps between the two genotypic cohorts and mQTLs were computed by including putative gene symbol matches for SNPs at a linkage disequilibrium value at least of 0.8 in individuals of European Ancestry included in the *1000 Genomes Project*, according to the *HaploReg* web tool (36). Finally, literature analysis was performed for duplicate findings (e.g., SNPs related to the same putative gene in at least two of the three lists: stringent mQTL list and any of the two SNP association lists) as described for mQTLs above.

### Regression models

Multivariate regression models with genotypic and transcriptomic data were regressed on phenotypic information. A vector was created containing the Spearman’s correlation of the WHO Meningioma Grade distribution with each module probe, hereafter called *Gene Significance for WHO Meningioma Grade* (*GS WHO_Grade*). A analogous vector containing the Spearman’s correlation of each module probe with each mQTL, was designated GSmQTL. Intramodular connectivity (kIN) was defined as the sum of the absolute value of the Spearman’s correlation of each probe with all the other probes in the same module. Another measure of connectivity, known as kME or MM, was calculated as previously defined in Section “Gene-Expression Analysis.”

Regression analysis was performed with the *lm* function, implemented in the package *stats* (21). A stepwise selection of models based on Akaike information criterion (AIC) was conducted. Significant variables were selected for further analysis if their *P*-value in the best AIC model was in the order of 10^−3^ or below, according to a *T* test. Rank transformation into normality function [*rntransform*, package *GenABEL* (45)] was applied to the dependent variables of the models. Normality of the dependent variables and the residuals was assessed with the function *shapiro.te*st (package *stats*). Variance inflation factors (VIFs) for lineal models were calculated with the *VIF* function implemented in the package *fmsb* (46).

### Miscellaneous

Boxplots present in Figure 3C, Figures S1A and S2E in Supplementary Material were created with the “*boxplot*” function implemented in the *graphics* package (21), and they represent the upper and lower quartiles (upper and lower of the boxes), the median (horizontal line inside the boxes), as well as the most extreme values in the dataset (the whiskers and the points outside it). Note that the whiskers include dispersed data that fall within 1.5 times the height of the box, and points represent values lying outside this range. Expression missing values were imputed with functions of the package *impute* (47).

## Results

### Co-expression analysis

**Figure 2:**
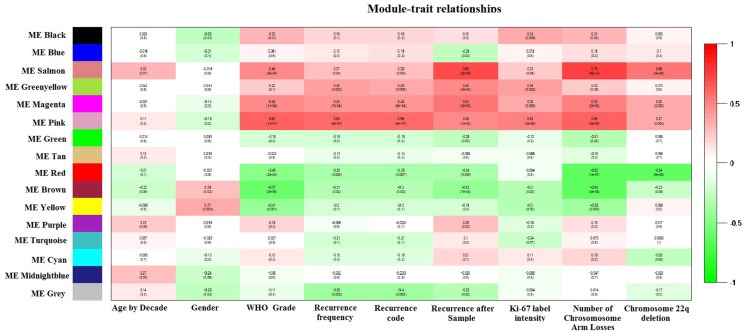
**Module–trait relationships plot**. Spearman’s correlation between module principal components (a.k.a module eigengenes, MEs) and *Age by decade* (first column), *Gender* (second column), *WHO Meningioma classification* (third column), *recurrence frequency* (fourth column), *recurrence code* (recurrent vs. newly diagnosed, fifth column), *recurrence after sample* (sixth column), *maximum Ki-67 step function* (absent = 0, low = 1, medium = 2, high = 3; seventh column), *sum of chromosome arm losses* (eighth column), and *Chromosome 22p deletion* (ninth column) is shown.

Weighted gene co-expression network analysis identified 16 co-expression modules. Module–trait relationships revealed that the pink ME was highly and significantly correlated with WHO Meningioma Grade, recurrence frequency, observed recurrence after sample, *Ki-67* staining, and with the total sum of chromosome arm losses (Figure 2). Boxplots of mean gene significance with WHO_Grade revealed that the Pink-module contains the most correlated genes (Figure S1A in Supplementary Material). *MM* (*a.k.a kME*) to the sixth power was determined to be in a marked 0.88 Spearman’s correlation (*P*-value <10^−16^) with *intramodular connectivity* (*kIN*), which is indicative of its usefulness to study high-level modular network properties. MM vs. GS WHO_Grade (Figure S1B in Supplementary Material) revealed a 0.72 Spearman’s correlation value (*P*-value ≤10^−16^), showing that genes importantly associated with disease stage are also the more relevant in the module. Pink-module gene-expression standard deviation was found to be inversely correlated with kIN (Spearman’s *rho* = −0.06, one-sided *P*-value = 0.09), but curiously higher levels of expression variability co-exist with low levels of connectivity (Figure S1C in Supplementary Material). For example, genes with a kIN below 2 are significantly more variable than those whose kIN value is above 2 (Wilcoxon Rank Sum Test *P*-value = 0.002, 95% C.I. = 7.74–36.96). This is consistent with the role of hub genes as central players in complex biological phenotypes (48). Moreover, almost 76% of the pink-module genes found in the replication cohort were significantly differentially expressed between tumor stages (FDR-adjusted *P*-value <0.05), and differentially expressed genes had a significantly greater connectivity (MM or kME) than the overall group of pink-module genes (Wilcoxon Rank Sum Test *P*-value = 0.043, 95% C.I. = 6.54 × 10^−5^–3.70 × 10^−2^).

**Figure 3:**
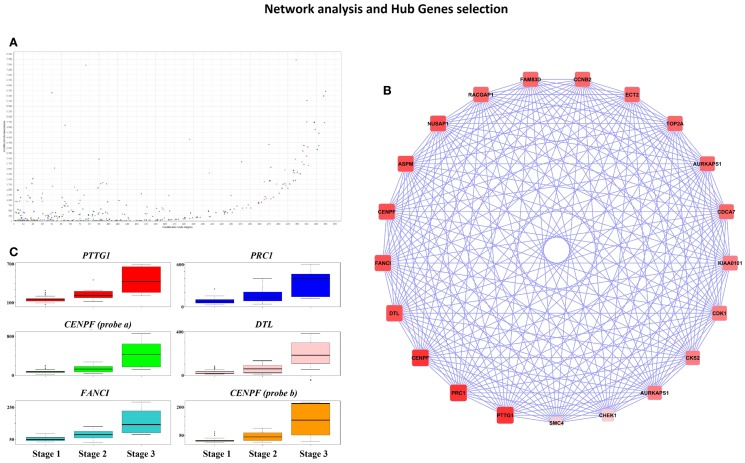
**(A)** *CentiScape* Plot of Pink-module network degree and betweenness values shows a tendency for highly connected genes to be also major avenues of communication among all the network components. **(B)** High degree (value >250) and betweenness genes (value >2,500) were selected as hub genes. The circular plot shows genes ordered according to their degree value, so that darker red nodes have higher values that lighter red ones. **(C)** Boxplots representing gene expression for the top six hub genes in each of the three WHO Grade meningioma groups. Represented genes are *PTTG1* (upper left, red), *PRC1* (upper right, blue), *CENPF* (probe 207828_s_at; middle left, green color), *DTL* (middle right, pink), *FANCI* (lower left, turquoise), and *CENPF* (probe 209172_s_at, lower right, orange).

**Figure 4:**
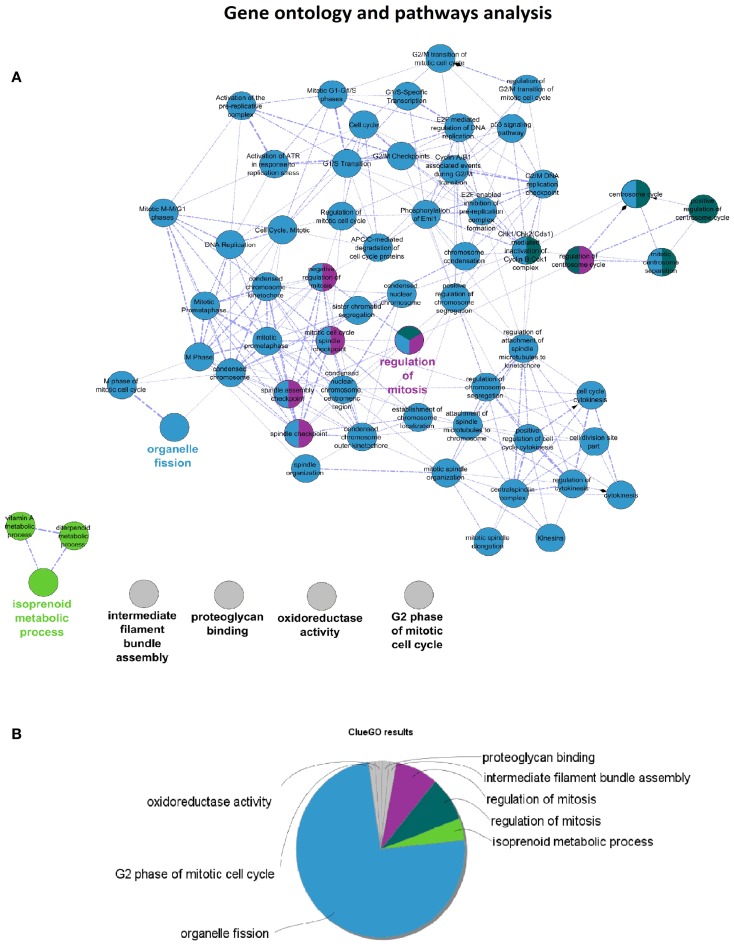
**(A)** *ClueGO* results network for Pink-module analysis. This network is created with Cohen’s kappa statistics, reflecting similarity between biological terms based on the similarity of their associated genes. **(B)** *ClueGO* results pie chart of the same data. After the analysis, eight clusters of biological terms, labeled with different colors, were obtained. Prevalence of cell-cycle related processes is visually obvious.

*Cytoscape* analysis of the network was used to choose the hub genes based on their *degree* and *betweenness* values in the network (Figure 3A). All hub genes were positively correlated with WHO_Stage (Figure 3B and in Table S4 in Supplementary Material), and graphical information about the top six can be consulted in Figure 3C. Furthermore, a stringent search for *Gene Ontology* and *Pathway* terms enrichment using *ClueGO* revealed a marked predominance in cell-cycle pathways. *Isoprenoid metabolic processes, intermediate filament bundle assembly, proteoglycan binding*, and *oxidoreductase activity* terms also passed the cutoff (Figures 4A,B). *DAVID* ontology analysis for hub genes revealed a marked enrichment in processes related to microtubule cytoskeleton. This is remarkably true for spindle and chromosome-related microtubular processes (Table S5 in Supplementary Material).

### Determination of mQTLs

**Figure 5:**
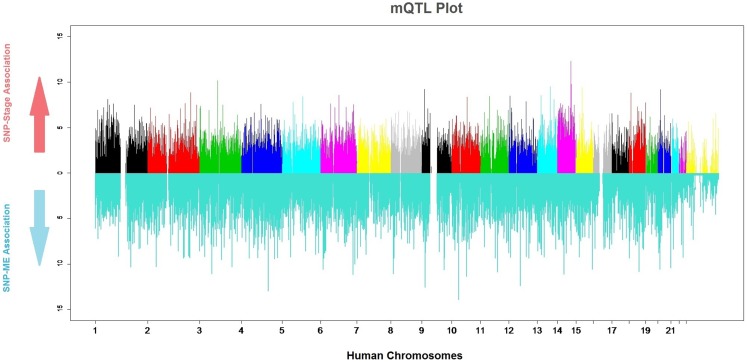
**mQTL plot**. The colored upper part of the plot indicates the association of each SNP with meningioma stages (0: meningioma WHO Grade I, 1: meningioma WHO Grade II and III). The significance value is expressed as −*log (P-value)*. The lower, blue part of the plot indicates the strength of the association of each SNP and the module.

Genomic markers and gene-expression data were merged into a single study to analyze quantitative relationships between them (QTLs). Due to the established relationships between several meningioma characteristics and the genes in the Pink-Module, we sought to determine genetic loci particularly affecting its expression (*mQTLs*, Figure 5). By fitting logistic regression models, we found 48 SNPs associated with the ME at *P*-values in the range of 10^−5^ or less. Four of these had *P*-values in the range of 10^−6^ and one was on the range of 10^−7^ (Table S2A in Supplementary Material). These SNPs correspond to four genomic loci mapping to the *ANKRD50, LOC389705, NRP1*, and *HOXC13* genes. To test possible improvements to this model, we performed logistic regression where the ME was included in the base model and significance for association with PC2 and PC1 + PC2 was tested (Tables S2B,C in Supplementary Material). The results indicated at an SNP in the *GRIN3A* gene with a *P*-value of roughly 0, and others at the *IBTK, ANGPT1, ASTN2*, and *LPIN2* genes with *P*-value in the range of 10^−6^ or below. Furthermore, logistic regression with PC1 + PC2 as regressor discovered 59 SNPs in 52 different genes showed *P*-values in the range of 10^−5^ or less (Table S2D in Supplementary Material). Nine of the SNPs had *P*-values in the range of 10^−6^ and were located at or near to *LOC339535, FMN2, NEK7, IL6R, C14orf64, KRT72*, and *SPRY2*. Five of them had *P*-values in the range of 10^−7^ and were located at or near to *ATF6, LOC100288079, USH2A, SUCLG2*, and *PHC1*. Finally, eight SNPs were on the range of 10^−8^ mapping in or close to *DNAJC19, HSP90AA4P, CHD10, XKR6, CA1, RASSF9, APAF1*, and *LOC100130792*.

We made a search in PubMed for the known implication of all putative mQTL genes in cancer and meningioma. More than half of the genes (63 out of 108, 58%) have supporting evidence for their direct implication in cancer, whilst only 5 out of 108 (5%) are known to be involved in meningioma pathogenesis. This difference is not surprising, since meningioma constitutes a less intensive research field than other cancers. The latter genes are *ALCAM, NRP1, IGF1R, CA1*, and *ERCC4*. Of these, *NRP1* and *IGF1R* do not have a clearly related role in meningioma yet, but evidence points toward the implication of the former in meningioma-associated neoangiogenesis (49) and for the blocking of the latter as therapeutic option (50).

### Gene ontology, pathway, protein–protein interaction, miRNA target site, and TFBS enrichment analysis of mQTL genes

A search for miRNA sets enriched in *mQTL* proximal genes was performed using GSEA, which revealed a significant enrichment in 36 of the sets (FDR-adjusted *P*-value <0.05). The two top findings were *MIR-124A* and *MIR-34B*, both with FDR-adjusted *P*-values in the order of 10^−5^ (Table S2E in Supplementary Material).

Enrichment for gene ontology terms and pathways associated with mQTL genes using ClueGo revealed enrichment in adherens junction interactions and cell–cell junction organization, response to low-density lipoproteins and cholesterol storage, endothelial cell proliferation, sprouting angiogenesis, negative regulation of vascular permeability, calcium-channel activity, NMDA glutamate receptors, actin and myosin-related pathways, meiotic chromosome segregation, protein localization to mitochondrion, and oxidoreductase activity (Table S2F in Supplementary Material). All terms above showed *FDR*-adjusted *P*-values <0.05.

STRING database was used to detect known interactions between all putative *mQTL* genes at the PPI level. Using a high confidence interaction score, 9 interactions were detected between the 87 proteins present in the database. Only 2.96 interactions were expected under the null hypothesis, thus concluding that the mQTL PPI network is significantly enriched in interactions (*P*-value <0.0035). Five of the interactions were among a cluster of four cadherin proteins (namely *CDH12, CDH13, CDH8*, and *CDH9*).

Transcription factor binding site (TFBS) enrichment was performed by including all mQTL putative genes in a search with *oPPOSUM Human Single Site Analysis* web tool. A marked significant enrichment in the Forkhead group of TFBS (FOXD3, FOXF2, FOXI1, FOXQ1, FOXA2, FOXD1), and in the Homeo group (LHX3, NOBOX, PDX1, PRRX2, NKX2-5, NKX3-1, and PBX1) was observed (Table S2G in Supplementary Material).

### SNP association with tumor stage

Tests for association between SNPs and tumor stage encoded as a binomial factor [either lowly malignant (stage I) or malignant (stages II and III)] on 84 subjects resulted in 5 SNPs ranking with a *P*-value on the order of 10^−05^ and 2 on the order of 10^−06^ (Figure 5; Table S2H in Supplementary Material). These two of the SNPs were located in an intergenic region 600 kb 5′ to *SEL1L* (*rs1652605* and *rs1958666*). By applying the same method in a Spanish cohort to find duplicate meningioma stage associated regions, other six SNPs in the same intergenic region (*rs17669975, rs11159518, rs1198035, rs1210840, rs1953408*, and *rs1198030*) were also substantially associated with meningioma stage (Table S6 in Supplementary Material). Curiously, this region encodes a long non-coding RNA known as *RP11-666E17.1-001*, whose function is unknown.

Inflation values (λ) (51) were estimated to be 1.15 for the first cohort and 1.02 for the second (replication) cohort.

### In-module genes and mQTL putative genes

In the search for possible in-module genes that may play an important regulatory role at the genomic scale, we searched for in-module genes that mapped to any of the mQTL genes. *BTBD3, LOC339535*, and *TBC1D9* genes were present in both lists, and thus are potentially important in meningioma pathogenesis.

### Duplicate SNP-related gene findings

In addition to the SEL1L-related SNPs that we described above, we also found coincidences between other mQTL-related genes and the tumor stage association cohorts. By searching for SNPs related to the input genes in LD as described in the Section “Materials and Methods,” we could identify *SPRY2* as a gene with SNPs in strong linkage disequilibrium between the mQTL list and the first genotypic cohort, whilst overlaps were found between the mQTL list and the validation genotypic cohort at the *C3orf67, C6, MEOX2*, and *SLC39A10* loci.

### Multivariate regression analysis

Pink-module co-expression, mQTL, and meningioma phenotypic data were used to create multivariate regression models. In the first, 1 kME was regressed on GS WHO_Grade (*GS WHO_Grade* ~ *kME*), and an adjusted *R*^2^ value of 0.51 was retrieved (*P*-value <2.2 × 10^−16^, VIF = 2.04, *R* = 0.71; Figure S2A in Supplementary Material). Afterward, we regressed GSmQTL SNPs on GS WHO_Grade (*GS WHO_Grade* ~ *GSmQTLs*) stepwise for a minimum AIC, obtaining an adjusted *R2* value of 0.88 (*P*-value <2.2 × 10^−16^), but indicative of multicollinearity (VIF = 9.56). Stepwise regression by including kME and GSmQTLs variables (*GS WHO_Grade* ~ *kME* + *GSmQTLs*) reached an adjusted *R2* value of 0.89 was achieved (*P*-value <2.2 × 10^−16^), but again multicollinearity was observed (VIF = 10.49). We collected all significant variables (*T* test *P*-value <0.01) and divided them into two groups according to their estimates signs. The variables with negative estimates were summed into a vector known as GSmQTLProtective, whilst the variables with positive estimates were added to a vector known ad GSmQTLRisk.

GSmQTLRisk correlated strongly with GS WHO_Grade (Spearman’s rho = 0.79; *P*-value ≤2.2 × 10^−16^; Figure S2B in Supplementary Material) while *GSmQTLProtective* mildly correlated with GS WHO_Grade, but that correlation was significant (Spearman’s rho = 0.33; *P*-value = 4.2 × 10^−14^; Figure S2C in Supplementary Material). Modeling GS WHO_Grade with kME and these new variables instead of its originals led to a simplified model with very similar results: adjusted *R2* value of 0.82 (*P*-value <2.2 × 10^−16^, *R* = 0.91). All variables ranked significant (*P*-value <2.2 × 10^−16^), and multicollinearity was not observed (VIF = 5.57). Indeed, GS WHO_Grade prediction according to this model was very accurate to reality (Spearman’s rho = 0.91, *P*-value <2 × 10^−16^; Figure S2D in Supplementary Material).

Finally, *kME, GSmQTLProtective*, and *GSmQTL1Risk* were split by their medians to dichotomize the data. After plotting the results with GS WHO_Grade, two groups of four plots each were easily differentiable, revealing that genes with higher connectivity (labeled *k*+) tended to have higher correlation with WHO Grade (Figure S2E in Supplementary Material). This is consistent with the finding that WHO Grade distribution is deeply correlated with connectivity. It is notorious that genes with higher values of *GSmQTLProtective* tended to have more negative correlations with GS WHO_Grade, while the opposite happened with genes whose *GSmQTLRisk* were high. The groups of genes with high *kME* and *GSmQTLRisk* and low or high *GSmQTLProtective* values were the most correlated with GS WHO_Stage, whilst the group of genes with low values of *kME* and *GSmQTLRisk* and high values of *GSmQTLProtective* was the less correlated group with GS WHO_Stage. Thus, the highest absolute correlation with meningioma Grade is achieved by genes with high connectivity and high association to putatively protective mQTLs.

## Discussion

Using WGCNA, we demonstrated that meningioma tissue expression can be summarized to the existence of 16 co-expression modules. One of them was found to be highly correlated with disease progression parameters. In this network, gene expression was less variable for highly connected genes than for mildly connected ones and hub genes had a higher and significant tendency to be positively correlated with tumor stage. Such characteristics are suggestive of the implication of this module in core biological functions related to tumor progression. Moreover, a group of SNPs were associated to a list of genomic regions both at the “suggestive level” (i.e., *P*-value at 10^−5^ level) and at the “significant level” (*P*-value ≤10^−6^).

Ontology analysis of in-module genes revealed a very significant enrichment in terms related to cell-cycle pathways. Curiously, many cancer-related pathways ranked high among the results, especially isoprenoid metabolism, intermediate filament bundle assembly, proteoglycan binding, and oxidoreductase activity pathways. There is evidence pointing toward *isoprenoid regulation of cell growth* (52). Indeed, isoprenoid compounds are known suppressors of carcinogenic processes such as telomerase activity (53), as well as activators of cell-cycle arrest and apoptosis signals (54). Intracranial tumors are known to have an increased activity of the low-density lipoprotein receptor due to an increased requirement of cholesterol (55). In fact, there is a body of evidence suggesting that statins, which are inhibitors of the HMG-CoA reductase pathway (blocking isoprenoid synthesis), may benefit cancer therapeutics (56). *Intermediate filaments* are widely used as markers of cancer differentiation and prognosis (57, 58), while *proteoglycans* are known mediators of tumor cells interaction with their microenvironment and regulators of angiogenesis (59, 60). *Oxidoreductase activity* plays a role in cancer too, since a recent meta-analysis of 21 studies has shown that polymorphisms in the NAD(P)H quinine oxidoreductase 1 (*NQO1*) gene are significantly associated with digestive tract cancer risk among Europeans and Asians (61). Moreover, a careful analysis of the three network hub genes (*PTTG1, PRC1*, and *CENPF*) reveals that *PTTG1* is linked to tumor malignancy and has been proposed as a possible therapeutic target (62); *PRC1* encodes a factor responsible for polarizing parallel microtubules and concentrating the factors responsible for contractile ring assembly, and has been involved in the growth of breast cancer cells (63, 64); and that *CENPF* encodes a protein that associates with centromere and kinetochore complexes. *CENPF* amplification is a frequent event in hepatocellular carcinomas (65). These data are in concordance with previous studies where co-expression networks were found to be strongly enriched in specific functions (66–68).

A focused analysis of the top 21 hub genes revealed a marked enrichment in *microtubule-associated processes*, including *spindle* and *chromosome-related pathways*. Cell-cycle studies have demonstrated that the metaphase–anaphase transition depends on a correct attachment of sister chromatids to the kinetochore-microtubule apparatus, which is by itself a crucial checkpoint (known as *M-phase checkpoint*) that prevents progression until all chromosomes are correctly joined to the spindle (69). Mad1 and Mad2 are two proteins known to regulate this checkpoint, and alterations in their ratios are known to lead to chromosome instability and aneuploidy, thus promoting tumorigenesis (70). Indeed, during anaphase, the central spindle is formed, which consists of a bundle of microtubules interpolated between segregating chromosomes and permits normal cytokinesis. Curiously, PRC1 is essential for central spindle formation, although its interactions with other spindle proteins seem tangled and await further studies (71). Thus, it is tempting to speculate that M-phase checkpoint aberrations may play an important role in meningioma malignization.

Generally speaking, the pathway and ontology results for in-module genes are consistent with that of mQTL genes. For example, the enrichment of mQTL genes in actin and myosin-related pathways, oxidoreductase activity, chromosome segregation, and cholesterol metabolism pathways is in accordance with co-expression module results. Nevertheless, mQTL genes were also enriched in other cancer-related pathways. For example, five mQTL putative genes are part of the cadherin family, and at least two (*CHD12* and *CHD13*) have been broadly studied for their role in cancer (72–74). Genes related to angiogenesis and vascular permeability pathways had also significant matches, and at least one of them (*NRP1*) has been studied before in meningioma (49). NRP1 protein acts as a receptor of VEGF-A (75), and its blockade is under clinical research for various types to tumors (76). Indeed, the inhibition of angiogenic pathways with bevacizumab has been shown to have encouraging anti-tumoral effects in recurrent and progressing meningiomas (77). Calcium-channel activity, another significant finding in the list, is known to be involved in growth factor-mediated meningioma proliferation and the use of calcium-channel blockers has anti-proliferative and drug-sensitizing effects (78, 79). Two glutamatergic receptor genes were present in the mQTL list. These are *GRIN2A* and *GRIN3A*. The implication of both ionotropic and metabotropic glutamate receptors in carcinogenesis and cancer progression is well-known, especially in gliomas, melanomas, and breast and prostate cancers [reviewed in Ref. (80)]. Similarly, a region 5′ to *SEL1L* was the only duplicated gene significantly associated with meningioma progression in the two SNP cohorts. *SEL1L* is a tumor suppressor gene involved in the endoplasmic reticulum-associated degradation (ERAD) pathway, and known to be involved in the complex ER adaptations needed for the progression of multiple neoplasms (81, 82). Moreover, an SNP in the *ATF6* gene was significantly associated (*P*-value = 2.65 × 10^−7^) with the sum of the first and second module principal components. The protein encoded by this gene is a membrane glycoprotein with a key function in the ERAD pathway and acts as a sensor and transducer of the unfolded protein response. Interestingly, it has been recently shown that misfolded ATF6 needs SEL1L for degradation despite the transmembrane nature of ATF6 (83), which indicates that degradation pathways are much more diversified than expected in eukaryotes, but with a still unknown role in cancer biology.

mQTL genes are also enriched in TFBS of the *Homeobox* and *Forkhead* family of transcription factors, and indeed *FOXP1* and *HOXC13* are inside the mQTL list. The implication of both families of transcription factors in cancer development and progression is widespread, and they probably play important roles in meningioma malignancy too (84, 85). Similarly, a marked enrichment in *MIR-124A* and *MIR-34B* microRNAs was observed. This is in accordance to the known role of both genes in cancer-related pathways. For example, MIR-124A is correlated with breast cancer growth and aggressiveness (86), whilst MIR-34B, which has tumor-suppressive effects through the inhibition of *BCL-2* (87), is associated to renal cell cancer risk (88) and silenced in hepatocellular carcinoma (89).

The results indicate that the co-expression module as a whole is associated with a list of more than 100 SNPs genome-wide (here known as *mQTLs*), with *P*-values in the range of 10^−5^ or less. By integrating gene-expression network connectivity and the correlation of each module gene with WHO Meningioma Grade (*GS WHO_Grade*) and with mQTLs (GSmQTLs), a multivariate regression model was created with only three regressors that *explain almost 82% of the variability*. Overall, these findings indicate that mQTLs not only are associated with the module expression, but also add a significant amount of explained variability to the study models, reinforcing their biological importance.

The main limitation facing this pipeline is related to the short sample size of the genotypic cohorts. With the aim to overcome this problem, only disease-associated gene findings present in at least two of the three SNP lists created were considered. Moreover, the consistency of the findings with gene expression, PPI networks, miRNAs and TFBS target sites, pathway and ontology databases, and current scientific literature adds plausibility and supports the veracity of the final results. However, a word of caution must be added to the results, and this applies particularly to all gene-specific findings described above, since many of them need further experimental validation. It is the aim of our study to provide a framework of evidence for future research on the field of meningiomas.

## Conclusion

This study has evidenced that meningioma gene expression contains a co-expression module highly correlated with tumor malignancy, whose hub genes are markedly enriched in *microtubule-related pathways*. Genetic loci linked to cytoskeleton, cell–cell adhesion, angiogenesis, calcium-channels, glutamate receptors, and ERAD pathways were determined as potential regulators of the whole co-expression network. Integration of these data with alternative gene-expression data, PPI networks, TFBS and miRNA target sequence databases, and scientific literature confirmed the enrichment of mQTLs in functional cancer-related networks. Moreover, SNP association with meningioma stage evidenced that a gene of the *ERAD pathway, SEL1L*, is likely associated with the malignant transformation of meningiomas, consistent with mQTL, and state-of-the-art knowledge. To sum up, this paper presents for the first time an integrated view of gene expression and genomic markers in the field of meningiomas. This resulted in the discovery of several pathways involved in meningioma malignant conversion and paves the way for the development of future research lines that will shed light on meningioma biology and its treatment. The reader is encouraged to watch the video summary in Video S1 in Supplementary Material (https://www.youtube.com/watch?v=fSf9QVu_lmE&feature=youtu.be).

## Author Contributions

This study was written and directed by Adrián Mosquera Orgueira. José Carlos Iglesias Gómez took part in the development of the visual content of the study and in the interpretation of the results.

## Conflict of Interest Statement

The authors declare that the research was conducted in the absence of any commercial or financial relationships that could be construed as a potential conflict of interest.

## Supplementary Material

The Supplementary Material for this article can be found online at http://www.frontiersin.org/Journal/10.3389/fonc.2014.00147/abstract

Figure S1 **(A)** Boxplots showing average correlation of each module’s genes and WHO Meningioma classification. **(B)** Plot of Module Membership for each gene and Pearson’s correlation for WHO Meningioma classification. **(C)** Plot of pink-module gene-expression standard deviation vs. intramodular connectivity reflects that low connectivity genes are more variable.

Figure S2 **(A)** Plotting of connectivity (kME) vs. GS WHO_Grade reveals a significant (*P*-value <10^−20^) Spearman’s correlation of 0.73 between both variables. **(B)** Spearman’s correlation of GSmQTLRisk and GS WHO_Grade is significant (*P*-value <10^−20^) and particularly high (*R* = 0.79). **(C)** Spearman’s correlation of GSmQTLProtective and GS WHO_Grade is significant (*P*-value = 4.2 × 10^−14^) and has a value of 0.33. **(D)** Prediction of GS WHO_Grade based on a multivariate regression linear model (where kME, GSmQTLProtective, and GSmQTLRisk were regressed on GS WHO_Grade) reveals strong and significant Spearman’s correlations with observed GS WHO_Grade values. **(E)** Dichotomized plots by the medians of kME (*K*±), GSmQTLRisk (*R*±), and GSmQTLProt (*P*±) evidence of two big groups based on kME values. Genes with high connectivity were more markedly correlated with GS WHO_Grade than those with lower connectivity values.

Video S1 **Artistic video summarizing the important aspects of the study**. Link: https://www.youtube.com/watch?v=fSf9QVu_lmE&feature=youtu.be

Table S1 **Summary of the original data downloaded for the study, including the links to the expression and genotypic files and the phenotypic information**.

Table S2 **(A)** mQTL list of association between SNPs and the ME. **(B)** mQTL list of association between the second principal component of the co-expression module and the SNPs. The base model of this regression was calculated including the ME. **(C)** Similar to **(B)** but the regressor was the sum of the ME and the second principal component. **(D)** mQTL list of association between SNPs and the sum of the first and second principal components of the co-expression module. **(E)** miRNA target site enrichment results for mQTL putative genes. **(F)** Pathway and Gene Ontology enrichment of mQTL putative genes. **(G)** Results of TFBS enrichment in mQTL putative genes. **(H)** SNP association with meningioma Grade according to the discovery cohort.

Table S3 **Annotation of the pink-module probes according to the array manufacturer**.

Table S4 **Hub genes list and network information for each one (degree and betweenness values)**.

Table S5 **Cell compartment gene ontology of the network hub genes, according to *DAVID***.

Table S6 **Top significant results of SNP association with tumor Grade in the replication cohort**.
