# Supplementary figures and images for: An Integrative Analysis of Meningioma Tumors Reveals the Determinant Genes and Pathways of Malignant Transformation
Source: Front Oncol. 2014 Jun 23;4:147. doi: 10.3389/fonc.2014.00147 (PMC4066933; doi:10.3389/fonc.2014.00147)

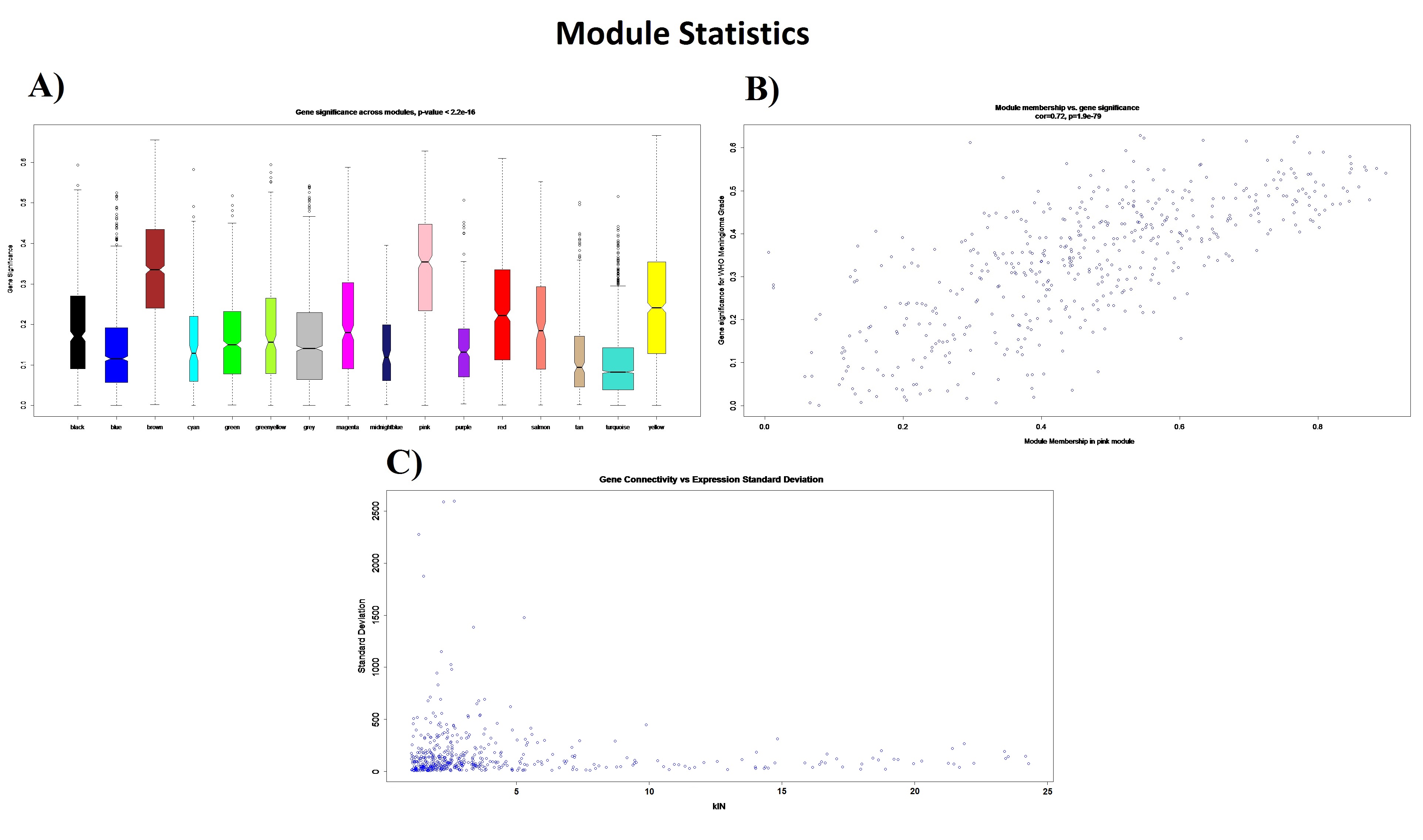

Supplement: Figure S1 — (A) Boxplots showing average correlation of each module’s genes and WHO Meningioma classification. (B) Plot of Module Membership for each gene and Pearson’s correlation for WHO Meningioma classification. (C) Plot of pink-module gene-expression standard deviation vs. intramodular connectivity reflects that low connectivity genes are more variable. [file Presentation1.ZIP › Supplementary Figure 1.jpg]

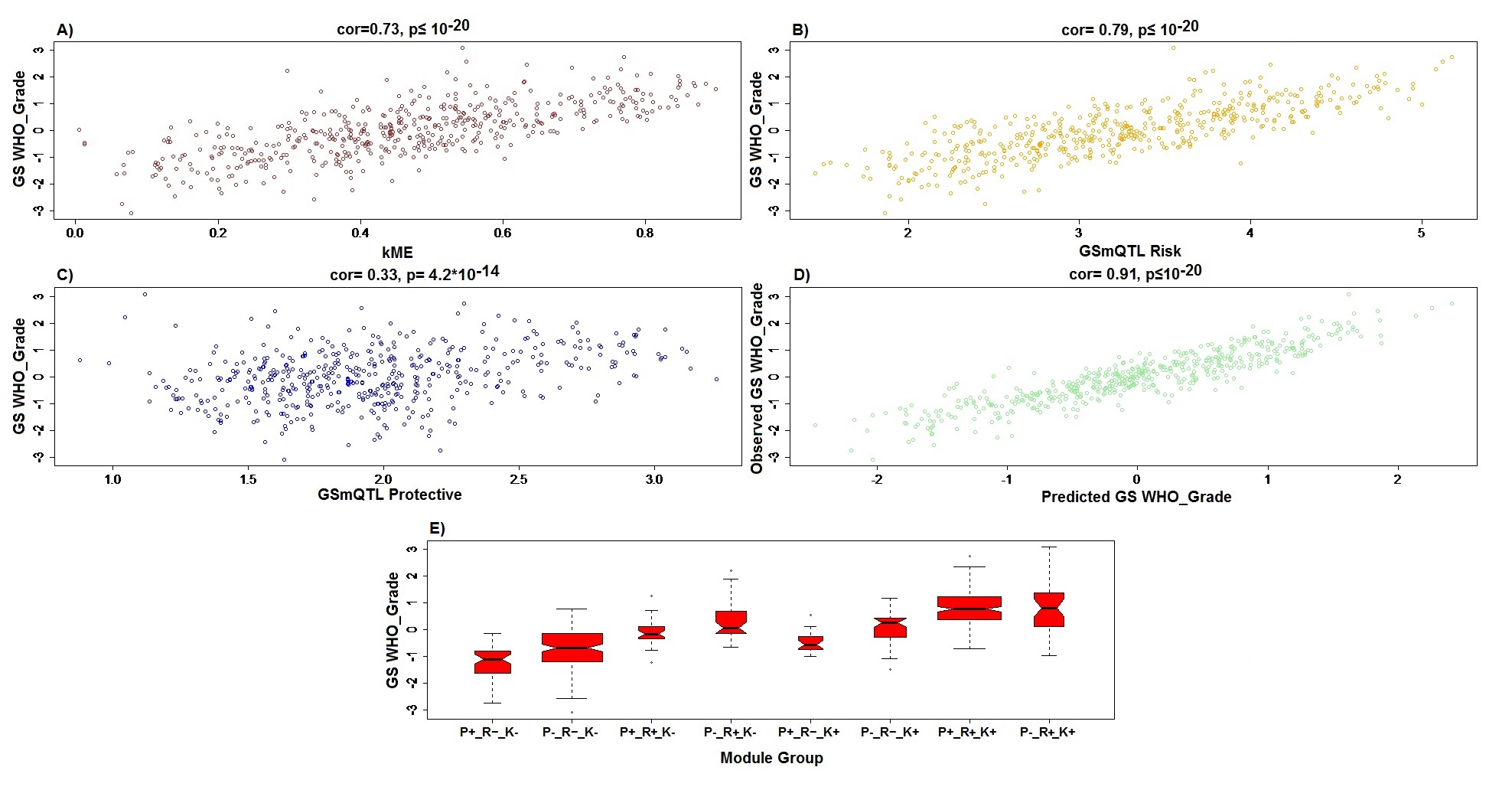

Supplement: Figure S1 — (A) Boxplots showing average correlation of each module’s genes and WHO Meningioma classification. (B) Plot of Module Membership for each gene and Pearson’s correlation for WHO Meningioma classification. (C) Plot of pink-module gene-expression standard deviation vs. intramodular connectivity reflects that low connectivity genes are more variable. [file Presentation1.ZIP › Supplementary Figure 2.jpg]
